# Dietary Factors and Female Breast Cancer Risk: A Prospective Cohort Study

**DOI:** 10.3390/nu9121331

**Published:** 2017-12-07

**Authors:** Ji Hyun Kim, Jeonghee Lee, So-Youn Jung, Jeongseon Kim

**Affiliations:** 1 Department of Cancer Control and Population Health, Graduate School of Cancer Science and Policy, National Cancer Center, 323 Ilsan-ro, Ilsandong-gu, Goyang-si 10408, Gyeonggi-do, Korea; 1601009@ncc.re.kr; 2 Department of Cancer Biomedical Science, Graduate School of Cancer Science and Policy, National Cancer Center, 323 Ilsan-ro, Ilsandong-gu, Goyang-si 10408, Gyeonggi-do, Korea; jeonghee@ncc.re.kr; 3 Center for Breast Cancer, National Cancer Center Hospital, National Cancer Center, 323 Ilsan-ro, Ilsandong-gu, Goyang-si 10408, Gyeonggi-do, Korea; goje1@ncc.re.kr

**Keywords:** food groups, dietary habits, dietary factors, Korean, breast cancer, prospective cohort study

## Abstract

Breast cancer is the leading cause of cancer in females and has become a major global health priority. This prospective cohort study investigated the association of dietary factors, including food items and dietary habits, with the risk of breast cancer in Korean women. Study participants were women aged 30 years or older, recruited from the National Cancer Center in South Korea between August 2002 and May 2007. They were followed until December 2014 using the Korea Central Cancer Registry to identify breast cancer cases. Among 5046 non-pre-diagnosed cancer participants, 72 breast cancer cases were prospectively identified. Participants with breast cancer had a significantly higher educational level (college or higher: 58.3% vs. 39.5%, *p* = 0.01), were more likely to have ever smoked (22.2% vs. 7.8%, *p* < 0.001), and were more likely to have a history of benign breast tumors (10% vs. 4%, *p* = 0.02) than non-cases. Consumption of grilled meat conferred a significantly higher risk of breast cancer in all women (hazard ratio (HR) 1.77, 95% confidence interval (CI) 1.09–2.85) and in postmenopausal women (HR 3.06, 95% CI 1.31–7.15). High-cholesterol food intake was associated with a higher risk in all women (HR 1.69, 95% CI 1.01–2.82). Irregular meal intake was associated with an elevated risk in all women (HR 2.19, 95% CI 1.20–3.98, *p* for trend = 0.01) and in premenopausal women (HR 2.35, 95% CI 1.13–4.91, *p* for trend = 0.03). Our findings suggest that grilled meat and high-cholesterol food intake and irregular eating habits may be associated with a higher risk of breast cancer. Further studies with longer follow-up periods that include information on portion size, hormone receptor status, carcinogen levels in grilled meat, and a classification of foods by source are required.

## 1. Introduction

Cancer is the leading cause of death worldwide and has become a major public health problem [1,2]. In Korea, cancer accounted for 28.3% of deaths in 2013 [3]. Of the various cancers, breast cancer is the most frequently diagnosed in women worldwide [1]. In Korea, the incidence of breast cancer has continually increased in the past 20 years [4] and was the second leading cause of female cancer in both incidence and prevalence in 2013. According to the Korean Central Cancer Registry (KCCR), the age-standardized incidence of breast cancer was 45.7 per 100,000 women, and the annual percent change between 1999 and 2013 was 5.9% in women [3].

Diet is considered a modifiable risk factor and accounts for approximately 35% of all cancer causes. Therefore, it is important to identify the dietary risk factors of cancer [5,6,7,8]. The westernization of Korea, including the rapid development and socioeconomic growth that have occurred since the 1950s, is speculated to have affected the incidence of cancer through drastic changes in lifestyle, including reproductive factors, physical activity, and diet [9,10,11,12]. Specifically, a rapid and unique transition in dietary patterns has emerged, as reflected by changes from the traditional diet based on grains and vegetables to a Western diet of predominantly meat and animal food products [13,14,15]. Vegetable intake may be inversely associated with cancer, but vegetables are generally consumed in pickled or salted forms in Korea, thereby increasing sodium intake, which is a dietary risk factor of cancer [16,17].

Previous epidemiological studies have addressed the dietary risk factors of breast cancer, considering nutrient or food items alone and together with dietary patterns [18,19,20,21,22,23,24]. Some studies found that higher intakes of alcohol [18], red and processed meat [19], and animal fat [20,21], and lower intakes of fruits and vegetables [22] and total dietary fiber [23], may be associated with a higher risk of breast cancer. Prudent/healthy dietary patterns reportedly decrease this risk, whereas no such evidence has been shown for Western/unhealthy diets [24]. However, the results have been inconsistent, and no clear association of breast cancer risk other than with alcohol consumption has been identified to date [7,25,26].

Dietary factors tend to differ by population due to socioeconomic status, ethnicity, and culture [27]. Therefore, risk factors for cancer in Korean populations may differ from those in Western countries, where most studies have been conducted [25,28]. Moreover, most previous studies in Korea on dietary risk factors of breast cancer were restricted to case-control studies; therefore, prospective cohort studies are needed to determine the exact associations [29].

Accordingly, the aim of this prospective cohort study was to investigate the association of dietary factors with the risk of breast cancer among all women and in groups stratified by menopausal status: premenopausal and postmenopausal women.

## 2. Materials and Methods

### 2.1. Study Population

**Figure 1:**
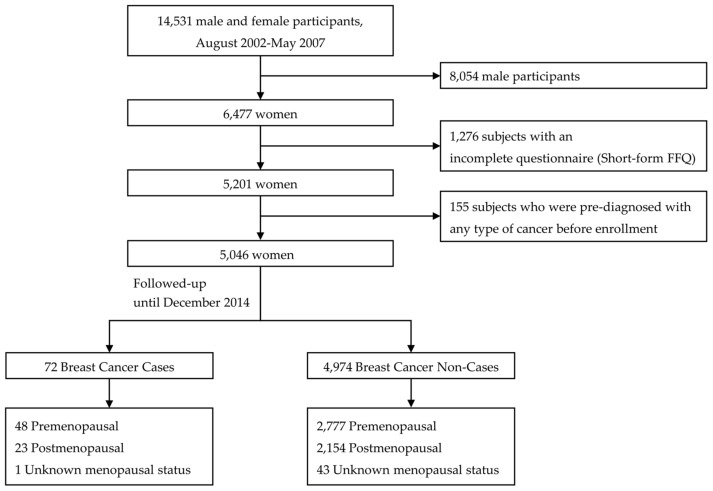
Flow chart of the study subjects.

The source population included 14,531 men and women aged 30 years and older enrolled at the Center for Cancer Prevention and detection at the National Cancer Center in South Korea between August 2002 And May 2007. Male subjects were excluded, resulting in the selection of 6477 female subjects. A total of 1276 subjects were excluded for having an incomplete short-form Food Frequency Questionnaire (FFQ), and 155 subjects were excluded due to a diagnosis with any type of cancer before enrollment. Potential breast cancer cases (International Classification of Diseases-10 Code C50) were ascertained by linkage to the 2014 KCCR database, which provides information on cancer incidence by following case classifications in Korea. after baseline examination of general characteristics and dietary factors (16 food groups and six dietary habits), the participants were followed up and contributed to the person-years until whichever came first: the date of any cancer diagnosis except for non-melanoma skin cancer (ICD-10 Code C44), death, or the end of the follow-up (December 2014). Seventy-two subjects developed breast cancer, and 4974 subjects were identified as breast cancer non-cases (Figure 1). Written informed consent was obtained from all participants, and the study protocol was approved by the institutional review board of the National Cancer Center (No. NCCNCS-07-077).

### 2.2. Data Collection and Management

At the time of enrollment, all participants were asked to complete self-administered questionnaires regarding socio-demographic characteristics (e.g., age, education, occupation, household income, and marital status), family history of cancer, cigarette and alcohol consumption, regular exercise habits, history of benign breast tumor, and reproductive factors (e.g., age at menarche, menopausal status, age at menopause, type of menopause, postmenopausal hormone use, parity, delivery frequency, lactation, and oral contraceptive use). Height and weight were measured using an InBody 3.0 (Biospace, Seoul, Korea) body composition analyzer or the X-SCAN PLUS II Body Composition Analyzer (Jawon Medical, Gyeongsan, Korea), and body mass index (BMI) was calculated as weight (kg)/height (m^2^).

The FFQ assessed 16 food groups: cereals, salty vegetables and seafood, light-colored vegetables, green-yellow vegetables, seaweed, fruit, grilled meat, healthy protein foods, dairy foods, bony fish, fried foods, high-cholesterol foods, animal fat-rich foods, sweet foods, fast foods, and caffeinated drinks (see Appendix A). The participants were asked to record the frequency of their current intake of each item according to eight categories: consumed rarely, once a month, two to three times a month, once a week, two to three times a week, four to six times a week, once a day, or more than two times a day. This FFQ has been used in several epidemiological studies on dietary factors related to chronic diseases [30,31,32] and health behaviors [33]. In our previous study, the FFQ was validated as a reference standard in 1401 participants from the source cohort based on a comparison with three-day dietary records. Cross-classification between the two methods of the distribution of subjects by tertiles indicated good agreement, ranging from 38% to 96% depending on the food group and nutrient [30].

Six questionnaires examining dietary habits were also assessed: meal frequency (three times a day, two times a day, irregular); breakfast frequency (always, often, not at all); meal time regularity (always regular, often regular, always irregular); meal speed (slow, average, fast); frequency of overeating (less than once a week, two to three times a week, more than four times a week); and the use of additional spices such as soy sauce, table salt, soybean paste, or hot pepper paste (often, sometimes, never).

### 2.3. Statistical Analysis

The characteristics of the study subjects were compared using Student’s *t*-test, chi-square test, or Fisher’s exact test. As the distribution of intake frequencies of each food item was skewed, the FFQ items were reclassified into two categories, low and high consumption, based on the median distribution of non-cases. A Cox proportional hazards model with person-years as the underlying time metric was used to evaluate the hazard ratio (HR) and 95% confidence interval (CI) of breast cancer for each dietary factor, including 16 food groups and six dietary habits. Multivariate Model 1 was adjusted for only significant factors of our study subjects’ general characteristics (Table 1), and Model 2 was adjusted for potential confounders according to other breast cancer researches [34,35]. Model 1 of all women was adjusted for age, smoking status (current, ex, non-smoker), education status (elementary school or less, middle school, high school, college or higher), and benign breast tumor history (yes, no). Premenopausal women were adjusted for age and smoking status (current, ex, non-smoker), and postmenopausal women was adjusted for age, education status (elementary school or less, middle school, high school, college or higher), and benign breast tumor history (yes, no). Model 2 was adjusted for age, BMI (<23.0, 23.0 to <25.0, ≥25 kg/m^2^), family history of breast cancer (yes, no), smoking status (current, ex, non-smoker), alcohol consumption (current, ex, non-drinker), physical activity (yes, no), age at menarche (≤13, 14, 15, ≥16 years), parity (0, 1, 2, ≥3), oral contraceptive use (yes, no), and benign breast tumor history (yes, no) in premenopausal women. In all women, we additionally adjusted for hormone use and menopausal status (premenopausal, postmenopausal hormone users, postmenopausal hormone non-users, unknown menopausal status), and age at menopause (premenopausal, menopause at <46, 46–48, 49–51, ≥52 years, unknown menopausal status). In postmenopausal women, we additionally adjusted for hormone use (yes, no) and age at menopause (<46, 46–48, 49–51, ≥52 years). All statistical analyses were performed using SAS software (version 9.4, SAS Institute, Cary, NC, USA), and the level of significance was set at *p* < 0.05.

## 3. Results

A total of 5046 Korean women were eligible for this analysis. During a mean follow-up of 9.46 years, 72 women were diagnosed with breast cancer. The subjects with breast cancer had a significantly higher educational level in the all group (college or higher: 58.3% vs. 39.5%, *p* = 0.01) and the postmenopausal group (college or higher: 56.5% vs. 270%, *p* = 0.04), were more likely to be ever-smokers in the all (22.2% vs. 7.8%, *p <* 0.001) and premenopausal groups (27.1% vs. 9.6%, *p <* 0.001), and more frequently had a history of benign breast tumor in the all (9.7% vs. 3.7%, *p* = 0.02) and postmenopausal groups (21.7% vs. 3.3%, *p* = 0.001) than did non-cases (Table 1).

Table 2 shows the intake frequencies of the 16 food groups based on the short-form FFQ and their association with breast cancer risk in all women. Consumption of grilled meat was associated with a significantly higher risk of breast cancer in the age-adjusted model, and this trend was maintained in multivariate Model 1 (HR 1.66, 95% CI 1.03–2.68) and Model 2 (HR 1.77, 95% CI 1.09–2.85). High-cholesterol food intake was positively associated with a higher risk only in the age-adjusted model (HR 1.71, 95% CI 1.03–2.85) and multivariate Model 2 (HR 1.69, 95% CI 1.01–2.82). Among the premenopausal women, there were no differences between the cases and non-cases (Table 3). In postmenopausal women, grilled meat consumption was associated with an elevated risk of breast cancer in both multivariate Model 1 (HR 2.41, 95% CI 1.05–5.54) and Model 2 (HR 3.06, 95% CI 1.31–7.15) (Table 4).

Table 5 shows the six dietary habits assessed and their associations with breast cancer risk in all women. Irregular meal intake was associated with an elevated risk of breast cancer in both multivariate Model 1 (HR 2.37, 95% CI 1.31–4.27, *p* for trend = 0.006) and Model 2 (HR 2.19, 95% CI 1.20–3.98, *p* for trend = 0.01). A similar finding regarding irregular meal intake was observed in premenopausal women in both multivariate Model 1 (HR 2.15, 95% CI 1.06–4.40, *p* for trend = 0.044) and Model 2 (HR 2.35, 95% CI 1.13–4.91, *p* for trend = 0.03) (Table 6), but not in postmenopausal women (Table 7).

## 4. Discussion

This prospective cohort study investigated the impact of dietary factors on the risk of breast cancer. After adjusting for confounding factors, grilled meat intake was proportionally associated with breast cancer risk in the all and postmenopausal groups, high cholesterol food intake was associated with a higher risk of breast cancer in all women, and meal irregularity was positively associated with breast cancer risk in the all and premenopausal groups.

The higher risk of breast cancer associated with grilled meat consumption is speculated to be due to carcinogenic mutagens such as heterocyclic amines (HCAs) and polycyclic aromatic hydrocarbons (PAHs), which are highly abundant in meat cooked at high temperatures, especially that which is grilled or barbecued [36,37]. HCAs are formed when creatinine, amino acids, and sugars present in meat muscles react at high temperatures [38]. PAHs are produced on or near the surface of meat when meat is cooked directly over an open flame and fat is pyrolyzed or when imperfectly combusted carbon and hydrogen from the fat fall onto hot coals and produce smoke [39]. One of the most common causes of PAH exposure in females is grilled food intake [40]. Although a few studies have shown a non-significant association of grilled meat consumption with breast cancer risk in both all women [41] and postmenopausal women [42], a similar trend to the one observed in this study was identified in several other studies. In case-control studies, grilled meat was associated with a higher risk of breast cancer in all women [43,44]. In postmenopausal women, a prospective study showed a borderline significantly higher breast cancer risk associated with the consumption of grilled/pan-fried/well-done meat [45], and a case-control study also showed a higher risk for total lifetime intake of grilled/barbecued beef, pork, and lamb [46]. The association of grilled meat with a higher risk of breast cancer in postmenopausal women may be due to differences in estrogen metabolism pathways based on menopausal status [41,47]. Before menopause, the ovaries are the major estrogen sites [48], whereas after menopause, adipose tissue plays a crucial role in synthesizing estrogen [47]. However, the mechanisms of the association between menopausal status and breast cancer and the interactions with diet remain unclear [49]. Fried foods also generate PAHs and HCAs [50], but they were not found to be associated with breast cancer risk in our studies. Previous studies showed inconsistent outcomes. One cohort study indicated that pan-fried meat was not significantly associated with breast cancer risk in postmenopausal women [42], although in other case-control studies, fried meat was associated with a higher risk in all women [43,44].

Other byproducts of grilled meat are advanced glycation end products (AGEs), which are created from a Maillard or browning reaction—non-enzymatic glycosylation of reducing sugars interacting with free amino groups of proteins, lipids, or nucleic acid [51,52]. By cross-linking with body proteins or binding with cell surface receptors, AGEs induce oxidative stress and inflammation and are therefore associated with dietary-related chronic diseases, including cancer [51]. $N^{\epsilon }$-carboxymethyllysine (CML) AGE is a biologically and chemically well-defined marker for analysis [53]. One study analyzed CML levels in 549 foods and concluded that, although AGEs are naturally present in animal foods, high-temperature cooking methods, especially frying, broiling, grilling, and roasting, produced more AGEs [51]. Some studies have used this database for analysis, and higher dietary CML was associated with pancreatic cancer in men [53], Alzheimer’s disease [54], and Barrett’s esophagus [55]. However, previous studies regarding breast cancer and AGEs have been limited to AGE receptor status [56,57] and the level of AGEs accumulated in breast tumors [58] to predict breast cancer progression rather than dietary AGE associations.

High-cholesterol food intake was associated with a higher breast cancer risk in the all women in the age-adjusted model and in multivariate Model 2, but this association was not found in multivariate Model 1. The role of dietary cholesterol in breast cancer remains unclear [59,60]. However, several in vivo and in vitro experiments have found that cholesterol may function as a signaling molecule in cancer cells as a cholesterol metabolite, 27-hydroxycholesterol (27HC), was found to act as an antagonist blocking estrogen receptor (ER) activation in the cardiovascular system and also functions as an agonist activating ER+ breast cancer [61]. Therefore, when cholesterol is converted to 27HC, it may induce ER+ breast cancer growth [61]. In several case-control studies, a study primarily consisting of postmenopausal women and two studies of all women showed that excessive cholesterol intake was associated with a higher risk of breast cancer [62,63,64]. In a cohort study, the baseline premenopausal women who remained premenopausal at the time of the last biennial questionnaire before censoring or the end of the follow-up also revealed that dietary cholesterol was associated with a higher risk of breast cancer [65]. However, in several cohort studies, no associations were found in all women [66,67,68] or postmenopausal women [69,70]. When the studies on dietary cholesterol and breast cancer risk were pooled in a meta-analysis, a negative association was found with breast cancer risk in all and premenopausal women [71].

In our study, the animal fat-rich food and fast food groups primarily consisted of processed meat products. Nitrate and nitrite, which are abundant in processed meats, are precursors of *N*-nitroso compounds (NOCs), which are potential carcinogens [72]. However, our study found no association between these food groups and breast cancer risk. Several previous studies also showed inconsistent trends. Processed meat intake was found to be associated with a higher risk of breast cancer among all women in case-control [41] and cohort studies [72,73], in postmenopausal women in nested case-control studies [74], and in both all women and in all subgroups of pre- and postmenopausal women in a cohort study [19]. By contrast, no association was observed between processed meat intake and the likelihood of breast cancer in all women in case-control [75] and cohort studies [76], in postmenopausal women in cohort studies [42,77], and in both all and pre- and postmenopausal women in cohort studies [78,79].

Light-colored, green-yellow, and salted vegetables and fruits were included in the category of fruits and vegetables. Although these food groups showed non-significant associations with the risk of breast cancer, several studies have indicated significant associations of foods other than salted vegetables. Vegetable intake may decrease the risk of breast cancer, but no association has been identified for fruits [22,80,81]. Similar results were reported in a meta-analysis of Korean studies as light-colored and green-yellow vegetable intake reduced the risk of breast cancer in all women, whereas fruit intake did not [29]. Among postmenopausal women reporting the highest lifetime intake of grilled/barbequed meat, those with the lowest intake of fruit and vegetables were associated with a significantly higher total lifetime risk [46]. However, a Korean case-control study found that the consumption of pickled vegetables was associated with a higher breast cancer risk in all women [81]. Other plant-based foods and soybean and its products were considered healthy protein foods. The intake of soy foods, including total soy products, soybean curd, and soymilk, showed a decreased risk of breast cancer in a Korean meta-analysis [29]. Our study observed no association of seafood, including seaweed and fish in the healthy protein food group and bonefish, with the risk of breast cancer. In a meta-analysis in Korea, a reduced risk of breast cancer was reported for seaweed, but not for fish consumption [29]. However, a case-control study in Korea identified a decreased risk with a combined vegetable–seafood dietary pattern [82].

We found no association between cereal consumption and breast cancer risk. Several cohort studies of postmenopausal women also showed non-significant associations of whole grain [83] and refined grain consumption [83,84], but conflicting results regarding bread and cereal were found in a case-control study [85]. Sweet foods also showed non-significant associations in our study, but other case-control studies reported different results. One study indicated a borderline significantly higher risk of early-stage breast cancer in the group with the highest intake of sweets [86]. Furthermore, another study that classified sources of sweets found that a higher intake of biscuits, sugar, and chocolate as food items and both desserts and sugars as a food group was associated with a higher risk of breast cancer and hypothesized that this was due to reduced diversity in the diet and increased meal frequency [87].

Dairy foods were not associated with breast cancer risk in our study. In a meta-analysis of 12 prospective studies, total dairy food intake was inversely associated with breast cancer, with low-fat dairy intake showing stronger associations in premenopausal women in particular [88]; however, a meta-analysis of Korean studies reported non-significant results [29]. The intake of caffeinated drinks also exhibited non-significant associations in our study. In a 22-year follow-up cohort study, no significant association of caffeinated drink intake with breast cancer risk was identified in all women, but a slightly reduced risk was observed in postmenopausal women [89].

Of the six dietary habits assessed, meal irregularity was associated with a higher risk of breast cancer in all and premenopausal women. However, no previous studies have addressed the direct association between regular eating and breast cancer risk. One study identified an interaction between eating frequency and an inflammatory biomarker, C-reactive protein (CRP), as a putative factor associated with breast cancer. This study showed that a higher eating frequency was inversely proportional to serum CRP levels, suggesting that eating more frequently may lower systemic inflammation and subsequently reduce breast cancer risk [90].

Adding spices to food was not associated with breast cancer risk. In a case-control study, adding table salt was a non-significant factor [75]. Psychological interruption may have affected this result. Subjects already recognize that a higher intake of sodium can increase the risk of chronic diseases, including cancer, and those who are concerned about their health may consequently select a lower frequency in the questionnaire. However, although the HR was not significant, the *P* for trend was significant in all women after adjusting for covariates. This result indicates that adding less spice may decrease the risk of breast cancer.

Our study has certain strengths: it is a cohort study with a follow-up period of more than nine years. We examined dietary factors at baseline when the subjects did not have breast cancer, so the reports were not subject to selection or recall bias. Additionally, as breast cancer is known to have a long latency, it is important to evaluate dietary patterns prior to the development of disease when environmental exposure may play a strong role [91], indicating that a cohort design would be more meaningful. Additionally, to our knowledge, this is the first prospective study conducted in Korea to address the associations between dietary habits and the risk of breast cancer.

Several limitations should also be mentioned: (1) Our FFQ data cover only baseline information and cannot reflect changes in dietary factors after baseline; (2) Additionally, residual confounding variables may have affected our results. Baseline nutritional status and genetic susceptibility may interact with diet and influence its relationship with breast cancer [25]; however, we could not adjust for these factors; (3) The food dataset included only frequency information, not portion size. Therefore, the intake amount could not be calculated, making it difficult to divide the study subjects into similar groups by the amount of intake of each food item; (4) Although the follow-up period was longer than nine years, there was still an insufficient number of breast cancer cases to infer strong statistical power; (5) The FFQ was limited to 16 items, making it difficult to ensure that all items of certain food groups were measured. Food items in a group may have different associations with breast cancer risk (e.g., red meat versus soy). A potential solution for this problem would be dividing the food group into corresponding food sources to specify their precise influences (e.g., rather than evaluating the consumption frequency of the healthy protein group, it is more appropriate to identify the intake frequency of each food item, such as soybean products, lean meat, fish, and egg whites); (6) Stratification of breast cancer by specific characteristics should be considered in further studies, especially hormone (estrogen and progesterone) receptor and human epidermal growth factor receptor 2 (HER-2) status [25].

## 5. Conclusions

In conclusion, this study showed that breast cancer risk was proportionally associated with grilled meat intake in all women and in postmenopausal women, with high-cholesterol food intake in all women, and with irregular eating in all and premenopausal women. Further studies with longer follow-up periods that include information on portion size, hormone receptor status, levels of each carcinogen in grilled meat, and classification of food groups by the source are needed.

## Figures and Tables

**Table 1:** General characteristics of the study subjects ^a^.

Characteristics	Total (*n* = 5046)			Premenopausal (*n* = 2825)			Postmenopausal (*n* = 2177)		
	Non-Cases (*n* = 4974)	Cases (*n* = 72)	*p* Value	Non-Cases (*n* = 2777)	Cases (*n* = 48)	*p* Value	Non-Cases (*n* = 2154)	Cases (*n* = 23)	*p* Value
**Age (years)**									
<40	1025 (20.6)	10 (13.9)	0.06	1000 (36.0)	10 (20.8)	0.09	15 (0.7)	0 (0.0)	0.33
40–49	1951 (39.2)	39 (54.2)		1599 (57.6)	34 (70.8)		336 (15.6)	5 (21.7)	
50–59	1390 (28.0)	14 (19.4)		177 (6.4)	4 (8.3)		1201 (55.8)	9 (39.1)	
60+	608 (12.2)	9 (12.5)		1 (0.0)	0 (0.0)		602 (28.0)	9 (39.1)	
**BMI (kg/m^2^)**									
<23.0	2692 (54.1)	41 (56.9)	0.89	1771 (63.8)	32 (66.7)	0.50	897 (41.6)	9 (39.1)	0.30
23.0–<25.0	1170 (23.5)	16 (22.2)		547 (19.7)	11 (22.9)		614 (28.5)	4 (17.4)	
≥25.0	1112 (22.4)	15 (20.8)		459 (16.5)	5 (10.4)		643 (29.9)	10 (43.5)	
**Body fat (%)**	29.21 ± 5.35	29.03 ± 5.73	0.78	27.88 ± 5.17	27.91 ± 5.52	0.99	30.88 ± 5.09	31.36 ± 5.67	0.66
**Education level**									
lementary school or less	529 (10.6)	3 (4.2)	0.01	79 (2.8)	0 (0.0)	0.42	449 (20.8)	3 (13.0)	0.04
Middle school	443 (8.9)	5 (6.9)		158 (5.7)	3 (6.3)		282 (13.1)	2 (8.7)	
High school	1957 (39.3)	21 (29.2)		1146 (41.3)	16 (33.3)		794 (36.9)	5 (21.7)	
College or higher	1962 (39.5)	42 (58.3)		1365 (49.2)	29 (60.4)		581 (27.0)	13 (56.5)	
**Occupation**									
Housewife	3045 (61.2)	40 (55.6)	0.33	1584 (57.0)	27 (56.3)	0.56	1443 (67.0)	13 (56.5)	0.37
Professional, office worker	740 (14.9)	16 (22.2)		537 (19.3)	12 (25.0)		192 (8.9)	4 (17.4)	
Sales, service	592 (11.9)	7 (9.7)		381 (13.7)	4 (8.3)		208 (9.7)	3 (13.0)	
Agriculture, labor, unemployed, other	463 (9.3)	8 (11.1)		223 (8.0)	5 (10.4)		236 (11.0)	3 (13.0)	
**Household income (10,000 won/month)**									
<200	768 (15.4)	6 (8.3)	0.06	270 (9.7)	3 (6.3)	0.10	494 (22.9)	3 (13.0)	0.42
200–<400	1386 (27.9)	16 (22.2)		807 (29.1)	8 (16.7)		569 (26.4)	8 (34.8)	
≥400	2226 (44.8)	41 (56.9)		1439 (51.8)	31 (64.6)		766 (35.6)	10 (43.5)	
**Marital status**									
Unmarried	165 (3.3)	3 (4.2)	0.90	147 (5.3)	2 (4.2)	0.93	17 (0.8)	1 (4.4)	0.06
Married	4378 (88.0)	62 (86.1)		2499 (90.0)	45 (93.8)		1847 (85.8)	17 (73.9)	
Divorced/widowed	384 (7.7)	6 (8.3)		112 (4.0)	1 (2.1)		267 (12.4)	5 (21.7)	
**Family history of breast cancer (Yes)**	168 (3.4)	4 (5.6)	0.31	91 (3.3)	2 (4.2)	0.67	76 (3.5)	2 (8.7)	0.21
**Smoking status**									
Current smokers	267 (5.4)	9 (12.5)	<0.001	196 (7.1)	7 (14.6)	<0.001	68 (3.2)	2 (8.7)	0.21
Ex-smokers	117 (2.4)	7 (9.7)		70 (2.5)	6 (12.5)		45 (2.1)	1 (4.4)	
Non-smokers	3847 (77.3)	48 (66.7)		2199 (79.2)	28 (58.3)		1617 (75.1)	19 (82.6)	
**Total pack-years**	0.7 ± 3.5	1.4 ± 4.2	0.22	0.7 ± 3.3	2.1 ± 5.2	0.13	0.6 ± 3.8	0.2 ± 0.7	0.03
**Alcohol consumption**									
Current drinkers	2300 (46.2)	36 (50.0)	0.80	1524 (54.9)	26 (54.2)	0.98	757 (35.1)	9 (39.1)	0.62
Ex-drinkers	188 (3.8)	2 (2.8)		108 (3.9)	2 (4.2)		79 (3.7)	0 (0.0)	
Non-drinkers	2224 (44.7)	31 (43.1)		1048 (37.7)	19 (39.6)		1157 (53.7)	12 (52.2)	
**Total alcohol consumption (g/day)**	17 ± 64	18 ± 49	0.93	22 ± 66	20 ± 55	0.86	11 ± 60	14 ± 35	0.78
**Physical activity (Yes)**	2242 (45.1)	35 (48.6)	0.86	1210 (43.6)	24 (50.0)	0.52	1011 (46.9)	11 (47.8)	>0.99
**Age at menarche (years)**									
≤13	981 (19.7)	13 (18.1)	0.88	749 (27.0)	10 (20.8)	0.66	228 (10.6)	3 (13.0)	0.67
14	963 (19.4)	16 (22.2)		631 (22.7)	14 (29.2)		325 (15.1)	2 (8.7)	
15	1111 (22.3)	14 (19.4)		651 (23.4)	11 (22.9)		450 (20.9)	3 (13.0)	
≥16	1587 (31.9)	22 (30.6)		625 (22.5)	10 (20.8)		955 (44.3)	12 (52.2)	
**Parity**									
0	328 (6.6)	7 (9.7)	0.20	257 (9.3)	5 (10.4)	0.96	69 (3.2)	2 (8.7)	0.10
1	467 (9.4)	7 (9.7)		353 (12.7)	6 (12.5)		110 (5.1)	1 (4.4)	
2	2587 (52.0)	43 (59.7)		1686 (60.7)	30 (62.5)		885 (41.1)	13 (56.5)	
≥3	1592 (32.0)	15 (20.8)		481 (17.3)	7 (14.6)		1090 (50.6)	7 (30.4)	
**Lactation (Yes)**	2903 (58.4)	43 (59.7)	0.84	1472 (53.0)	27 (56.3)	>0.99	1412 (65.6)	16 (69.6)	0.25
**Oral contraceptive use (Yes)**	864 (17.4)	12 (16.7)	0.91	403 (14.5)	7 (14.6)	>0.99	455 (21.1)	5 (21.7)	>0.99
**Benign breast tumor history (Yes)**	186 (3.7)	7 (9.7)	0.02	113 (4.1)	2 (4.2)	0.67	71 (3.3)	5 (21.7)	0.001
**Menopausal status (Yes) ^b^**	2154 (43.3)	23 (31.9)	0.07	-	-	-	-	-	-
**Postmenopausal hormone use (yes) ^c^**	-	-	-	-	-	-	613 (28.5)	9 (39.1)	0.31
**Age at menopause (years) ^c^**	-	-	-	-	-	-			
<46	-	-	-	-	-	-	380 (17.6)	4 (17.4)	0.53
46–<49	-	-	-	-	-	-	330 (15.3)	2 (8.7)	
49–<52	-	-	-	-	-	-	321 (14.9)	6 (26.1)	
≥52	-	-	-	-	-	-	488 (22.7)	5 (21.7)	
**Type of menopause ^c^**	-	-	-	-	-	-			
Natural	-	-	-	-	-	-	1485 (68.9)	17 (73.9)	0.84
Surgical, other	-	-	-	-	-	-	642 (29.8)	6 (26.1)	

Missing data are included in the total %. ^a^ The values are presented as the mean ± standard deviation (SD) or *n* (%) using Student’s *t*-test, chi-square test, or Fisher’s exact test; ^b^ In all women; ^c^ In postmenopausal women.

**Table 2:** Sixteen food groups and their associations with the risk of breast cancer in all subjects.

Food Group	Consumption Frequency ^a^	All Women										
		All (*n* = 5046)		Case (*n* = 72)		Person Years	Age-Adjusted		Multivariate 1 ^b^		Multivariate 2 ^c^	
		*N*	%	*n*	%		HR	95% CI	HR	95% CI	HR	95% CI
Cereals	Low	1738	34.4	26	36.1	16,876	1.00		1.00		1.00	
	High	3308	65.6	46	63.9	30,837	0.96	0.59–1.55	0.86	0.52–1.40	0.95	0.58–1.57
Salted vegetables and seafood	Low	2838	56.2	41	56.9	27,009	1.00		1.00		1.00	
	High	2208	43.8	31	43.1	20,704	0.98	0.62–1.57	0.99	0.62–1.58	0.98	0.61–1.58
Light-colored vegetables	Low	2083	41.3	32	44.4	19,610	1.00		1.00		1.00	
	High	2963	58.7	40	55.6	28,103	0.88	0.55–1.40	0.86	0.54–1.37	0.87	0.54–1.38
Green-yellow vegetables	Low	3101	61.5	38	52.8	29,564	1.00		1.00		1.00	
	High	1945	38.6	34	47.2	18,149	1.46	0.92–2.32	1.38	0.87–2.20	1.46	0.91–2.33
Seaweed	Low	1746	34.6	25	34.7	16,777	1.00		1.00		1.00	
	High	3300	65.4	47	65.3	30,936	1.01	0.62–1.64	0.96	0.59–1.57	1.06	0.65–1.73
Fruit	Low	2554	50.6	35	48.6	24,168	1.00		1.00		1.00	
	High	2492	49.4	37	51.4	23,545	1.09	0.69–1.73	1.05	0.65–1.70	1.22	0.76–1.97
Grilled meat	Low	2908	57.6	31	43.1	27,398	1.00		1.00		1.00	
	High	2138	42.4	41	56.9	20,315	1.80	1.12–2.91	1.66	1.03–2.68	1.77	1.09–2.85
Healthy protein foods	Low	2885	57.2	35	48.6	27,391	1.00		1.00		1.00	
	High	2161	42.8	37	51.4	20,322	1.42	0.89–2.25	1.25	0.78–2.00	1.46	0.91–2.34
Dairy foods	Low	2864	56.8	36	50.0	27,142	1.00		1.00		1.00	
	High	2182	43.2	36	50.0	20,571	1.33	0.83–2.10	1.24	0.78–1.97	1.32	0.83–2.11
Bony fish	Low	2293	45.4	31	43.1	21,766	1.00		1.00		1.00	
	High	2753	54.6	41	56.9	25,947	1.12	0.70–1.80	1.10	0.69–1.78	1.14	0.71–1.83
Fried foods	Low	2599	51.5	34	47.2	24,588	1.00		1.00		1.00	
	High	2447	48.5	38	52.8	23,125	1.18	0.73–1.89	1.06	0.66–1.71	1.19	0.74–1.92
High-cholesterol foods	Low	2153	42.7	22	30.6	20,527	1.00		1.00		1.00	
	High	2893	57.3	50	69.4	27,186	1.71	1.03–2.85	1.48	0.88–2.48	1.69	1.01–2.82
Animal fat-rich foods	Low	3143	62.3	43	59.7	29,641	1.00		1.00		1.00	
	High	1903	37.7	29	40.3	18,072	1.09	0.68–1.77	1.03	0.63–1.67	1.05	0.64–1.71
Sweet foods	Low	2180	43.2	32	44.4	20,395	1.00		1.00		1.00	
	High	2866	56.8	40	55.6	27,318	0.92	0.57–1.47	0.82	0.51–1.33	0.90	0.56–1.45
Fast-foods	Low	3152	62.5	42	58.3	29,741	1.00		1.00		1.00	
	High	1894	37.5	30	41.7	17,972	1.17	0.71-1.94	1.03	0.62-1.70	1.16	0.70-1.90
Caffeinated drinks	Low	2128	42.2	30	41.7	20,133	1.00		1.00		1.00	
	High	2918	57.8	42	58.3	27,580	1.01	0.63-1.62	0.88	0.55-1.42	0.90	0.55-1.46

Abbreviations are as follows: CI, confidence interval; HR, hazard ratio. ^a^ Cereals/Salted vegetables and seafood: low ≤ once a day, high ≥ 2 times a day. Light-colored vegetables/Caffeinated drink/Fruit: low ≤ 4–6 times a week, high ≥ once a day. Green-yellow vegetables/Healthy protein foods/Dairy foods: low ≤ 2–3 times a week, high ≥ 4–6 times a week. Seaweed/Bony fish: low ≤once a week, high ≥ 2–3 times a week. Grilled meat/High-cholesterol foods/Fast-foods: low ≤once a month, high ≥ 2–3 times a month. Fried foods/Animal fat-rich foods/Sweet foods: low ≤ 2–3 times a month, high ≥once a week; ^b^ Adjusted for age, smoking status (current, ex, non-smoker), education group (elementary school or less, middle school, high school, college or higher), breast benign tumor history (yes, no); ^c^ Adjusted for diverse potential confounders: total: age, BMI (<23.0, 23.0 to <25.0, ≥25 kg/m^2^), family history of breast cancer (yes, no), smoking status (current, ex, non-smoker), alcohol consumption (current, ex, non-drinker), physical activity (yes, no), age at menarche (≤13, 14, 15, ≥16 years), parity (0, 1, 2, ≥3), oral contraceptive use (yes, no), benign breast tumor history (yes, no), hormone use and menopausal status (premenopausal, postmenopausal users, postmenopausal nonusers, unknown menopausal status), and age at menopause (premenopausal, menopause at <46, 46–48, 49–51, ≥52 years, unknown menopausal status).

**Table 3:** Sixteen food groups and their associations with the risk of breast cancer in premenopausal women.

Food Group	Consumption Frequency ^a^	Premenopausal Women										
		All (*n* = 2825)		Case (*n* = 48)		Person Years	Age-Adjusted		Multivariate 1 ^b^		Multivariate 2 ^c^	
		*N*	%	*n*	%		HR	95% CI	HR	95% CI	HR	95% CI
Cereals	Low	929	32.9	15	31.3	9054	1.00		1.00		1.00	
	High	1896	67.1	33	68.8	17,666	1.15	0.63–2.13	1.19	0.64–2.20	1.15	0.61–2.17
Salted vegetables and seafood	Low	1560	55.2	25	52.1	14,877	1.00		1.00		1.00	
	High	1265	44.8	23	47.9	11,843	1.17	0.66–2.06	1.21	0.68–2.13	1.17	0.65–2.09
Light-colored vegetables	Low	1168	41.4	25	52.1	10,971	1.00		1.00		1.00	
	High	1657	58.7	23	47.9	15,749	0.63	0.36–1.10	0.64	0.36–1.12	0.61	0.35–1.09
Green-yellow vegetables	Low	1792	63.4	27	56.3	17,071	1.00		1.00		1.00	
	High	1033	36.6	21	43.8	9649	1.36	0.77–2.41	1.38	0.78–2.44	1.33	0.75–2.36
Seaweed	Low	940	33.3	19	39.6	9054	1.00		1.00		1.00	
	High	1885	66.7	29	60.4	17,666	0.78	0.44–1.40	0.81	0.45–1.44	0.76	0.42–1.38
Fruit	Low	1497	53.0	24	50.0	14,108	1.00		1.00		1.00	
	High	1328	47.0	24	50.0	12,612	1.10	0.62–1.94	1.25	0.71–2.22	1.23	0.69–2.20
Grilled meat	Low	1413	50.0	21	43.8	13,287	1.00		1.00		1.00	
	High	1412	50.0	27	56.3	13,433	1.36	0.77–2.42	1.33	0.75–2.36	1.36	0.77–2.43
Healthy protein foods	Low	1582	56.0	26	54.2	15,028	1.00		1.00		1.00	
	High	1243	44.0	22	45.8	11,692	1.08	0.61–1.91	1.12	0.63–1.97	1.12	0.63–2.00
Dairy food	Low	1666	59.0	26	54.2	15,786	1.00		1.00		1.00	
	High	1159	41.0	22	45.8	10,934	1.21	0.69–2.14	1.22	0.69–2.16	1.20	0.67–2.13
Bony fish	Low	1391	49.2	24	50.0	13,246	1.00		1.00		1.00	
	High	1434	50.8	24	50.0	13,474	0.92	0.52–1.63	0.96	0.54–1.70	0.95	0.53–1.69
Fried foods	Low	1222	43.3	22	45.8	11,554	1.00		1.00		1.00	
	High	1603	56.7	26	54.2	15,166	0.97	0.55–1.73	1.00	0.56–1.78	1.00	0.56–1.79
High-cholesterol foods	Low	1014	35.9	14	29.2	9707	1.00		1.00		1.00	
	High	1811	64.1	34	70.8	17,013	1.45	0.78–2.70	1.46	0.78–2.73	1.42	0.75–2.67
Animal fat-rich foods	Low	1519	53.8	27	56.3	14,284	1.00		1.00		1.00	
	High	1306	46.2	21	43.8	12,436	0.94	0.53–1.67	0.93	0.52–1.64	0.93	0.52–1.67
Sweet foods	Low	1036	36.7	18	37.5	9633	1.00		1.00		1.00	
	High	1789	63.3	30	62.5	17,087	1.01	0.56–1.82	1.07	0.59–1.94	1.02	0.56–1.86
Fast-foods	Low	1422	50.3	25	52.1	13,376	1.00		1.00		1.00	
	High	1403	49.7	23	47.9	13,344	1.03	0.58–1.85	1.07	0.60–1.92	1.05	0.58–1.89
Caffeinated drinks	Low	995	35.2	16	33.3	9373	1.00		1.00		1.00	
	High	1830	64.8	32	66.7	17,347	1.08	0.59–1.97	1.06	0.58–1.93	1.07	0.58–1.96

Abbreviations are as follows: CI, confidence interval; HR, hazard ratio. ^a^ Cereals/Salted vegetables and seafood: low ≤ once a day, high ≥ 2 times a day. Light-colored vegetables/Caffeinated drinks/Fruit: low ≤ 4–6 times a week, high ≥ once a day. Green-yellow vegetables/Healthy protein food/Dairy food: low ≤ 2–3 times a week, high ≥ 4–6 times a week. Seaweed/Bony fish: low ≤ once a week, high ≥ 2–3 times a week. Grilled meat/High-cholesterol foods/Fast-foods: low ≤ once a month, high ≥ 2–3 times a month. Fried foods/Animal fat-rich foods/Sweet foods: low ≤ 2–3 times a month, high ≥once a week; ^b^ Adjusted for age, smoking status (current, ex, non-smoker); ^c^ Adjusted for diverse potential confounders: total: age, BMI (<23.0, 23.0 to <25.0, ≥25 kg/m^2^), family history of breast cancer (yes, no), smoking status (current, ex, non-smoker), alcohol consumption (current, ex, non-drinker), physical activity (yes, no), age at menarche (≤13, 14, 15, ≥16 years), parity (0, 1, 2, ≥3), oral contraceptive use (yes, no), and benign breast tumor history (yes, no).

**Table 4:** Sixteen food groups and their associations with the risk of breast cancer in postmenopausal women.

Food Group	Consumption Frequency ^a^	Postmenopausal Women										
		All (*n* = 2177)		Case (*n* = 23)		Person Years	Age-Adjusted		Multivariate 1 ^b^		Multivariate 2 ^c^	
		*N*	%	*n*	%		HR	95% CI	HR	95% CI	HR	95% CI
Cereals	Low	786	36.1	11	47.8	7593	1.00		1.00		1.00	
	High	1391	63.9	12	52.2	12,986	0.62	0.28–1.41	0.54	0.24–1.23	0.53	0.22–1.25
Salted vegetables and seafood	Low	1254	57.6	16	69.6	11,894	1.00		1.00		1.00	
	High	923	42.4	7	30.4	8685	0.59	0.24–1.44	0.59	0.24–1.42	0.45	0.18–1.14
Light-colored vegetables	Low	894	41.1	7	30.4	8437	1.00		1.00		1.00	
	High	1283	58.9	16	69.6	12,142	1.59	0.65–3.86	1.38	0.56–3.36	1.37	0.55–3.39
Green-yellow vegetables	Low	1280	58.8	11	47.8	12,215	1.00		1.00		1.00	
	High	897	41.2	12	52.2	8364	1.58	0.70–3.57	1.34	0.59–3.07	1.42	0.62–3.30
Seaweed	Low	790	36.3	6	26.1	7566	1.00		1.00		1.00	
	High	1387	63.7	17	73.9	13,013	1.63	0.64–4.15	1.40	0.55–3.59	1.73	0.67–4.50
Fruit	Low	1035	47.5	10	43.5	9859	1.00		1.00		1.00	
	High	1142	52.5	13	56.5	10,720	1.18	0.52–2.70	0.86	0.36–2.03	1.22	0.51–2.92
Grilled meat	Low	1475	67.8	10	43.5	13,912	1.00		1.00		1.00	
	High	702	32.3	13	56.5	6667	2.80	1.22–6.41	2.41	1.05–5.54	3.06	1.31–7.15
Healthy protein foods	Low	1275	58.6	9	39.1	12,093	1.00		1.00		1.00	
	High	902	41.4	14	60.9	8486	2.23	0.96–5.15	1.77	0.74–4.20	2.28	0.94–5.52
Dairy foods	Low	1175	54.0	10	43.5	11,130	1.00		1.00		1.00	
	High	1002	46.0	13	56.5	9449	1.53	0.67–3.49	1.31	0.57–3.01	1.56	0.67–3.65
Bony fish	Low	882	40.5	7	30.4	8324	1.00		1.00		1.00	
	High	1295	59.5	16	69.6	12,255	1.53	0.63–3.72	1.34	0.54–3.29	1.38	0.55–3.46
Fried foods	Low	1351	62.1	11	47.8	12,790	1.00		1.00		1.00	
	High	826	37.9	12	52.2	7789	1.84	0.81–4.17	1.47	0.64–3.38	1.78	0.75–4.21
High-cholesterol foods	Low	1124	51.6	8	34.8	10,677	1.00		1.00		1.00	
	High	1053	48.4	15	65.2	9902	2.06	0.87–4.88	1.59	0.66–3.82	1.97	0.81–4.80
Animal fat-rich foods	Low	1595	73.3	16	69.6	15,080	1.00		1.00		1.00	
	High	582	26.7	7	30.4	5499	1.25	0.51–3.07	1.11	0.45–2.70	1.18	0.47–2.99
Sweet foods	Low	1121	51.5	13	56.5	10,552	1.00		1.00		1.00	
	High	1056	48.5	10	43.5	10,027	0.83	0.36–1.90	0.69	0.30–1.60	0.66	0.27–1.57
Fast-foods	Low	1699	78.0	16	69.6	16,078	1.00		1.00		1.00	
	High	478	22.0	7	30.4	4501	1.68	0.68–4.14	1.29	0.52–3.20	1.47	0.58–3.71
Caffeinated drinks	Low	1118	51.4	14	60.9	10,617	1.00		1.00		1.00	
	High	1059	48.6	9	39.1	9962	0.70	0.30–1.64	0.61	0.26–1.42	0.56	0.23–1.35

Abbreviations are as follows: CI, confidence interval; HR, hazard ratio. ^a^ Cereals/Salted vegetables and seafood: low ≤ once a day, high ≥ 2 times a day. Light-colored vegetables/Caffeinated drinks/Fruit: low ≤ 4–6 times a week, high ≥once a day. Green-yellow vegetables/Healthy protein foods/Dairy foods: low ≤ 2–3 times a week, high ≥ 4–6 times a week. Seaweed/Bony fish: low ≤once a week, high ≥ 2–3 times a week. Grilled meat/High-cholesterol foods/Fast-foods: low ≤once a month, high ≥ 2–3 times a month. Fried foods/Animal fat-rich foods/Sweet foods: low ≤ 2–3 times a month, high ≥ once a week; ^b^ Adjusted for age, education group (elementary school or less, middle school, high school, college or higher), breast benign tumor history (yes, no); ^c^ Adjusted for diverse potential confounders: total: age, BMI (<23.0, 23.0 to <25.0, ≥25 kg/m^2^), family history of breast cancer (yes, no), smoking status (current, ex, non-smoker), alcohol consumption (current, ex, non-drinker), physical activity (yes, no), age at menarche (≤13, 14, 15, ≥16 years), parity (0, 1, 2, ≥3), oral contraceptive use (yes, no), benign breast tumor history (yes, no), hormone use (yes, no), and age at menopause (<46, 46–48, 49–51, ≥52 years).

**Table 5:** Six dietary habits and their associations with breast cancer risk in all subjects.

Dietary Habit	Frequency	All Women										
		All (*n* = 5046)		Case (*n* = 72)		Person Years	Age-Adjusted		Multivariate 1 ^b^		Multivariate 2 ^c^	
		*N*	%	*n*	%		HR	95% CI	HR	95% CI	HR	95% CI
Meal frequency	3 times/day	3285	65.1	37	51.4	31,174	1.00		1.00		1.00	
	2 times/day	1110	22.0	18	25.0	10,327	1.49	0.84–2.63	1.37	0.77–2.44	1.36	0.76–2.42
	Irregular ^a^	616	12.2	17	23.6	5896	2.47	1.38–4.41	2.37	1.31–4.27	2.19	1.20–3.98
	*p* for trend						0.003		0.006		0.01	
Breakfast frequency	Always	2792	55.3	31	43.1	26,361	1.00		1.00		1.00	
	Often	1579	31.3	29	40.3	14,992	1.67	1.00–2.80	1.55	0.92–2.60	1.56	0.93–2.63
	Not at all	657	13.0	12	16.7	6193	1.68	0.85–3.33	1.41	0.71–2.84	1.43	0.71–2.90
	*p* for trend						0.06		0.18		0.17	
Meal time regularity	Regular	1969	39.0	36	50.0	18,474	1.00		1.00		1.00	
	Often	2549	50.5	25	34.7	24,404	0.52	0.31–0.87	0.53	0.32–0.90	0.52	0.31–0.88
	Irregular	510	10.1	11	15.3	4661	1.18	0.59–2.34	1.24	0.62–2.51	1.02	0.50–2.06
	*p* for trend						0.40		0.48		0.29	
Meal speed	Slow	557	11.0	7	9.7	5240	1.00		1.00		1.00	
	Average	2634	52.2	40	55.6	24,817	1.22	0.55–2.72	1.41	0.63–3.16	1.35	0.60–3.04
	Fast	1850	36.7	25	34.7	17,611	1.07	0.47–2.49	1.27	0.55–2.95	1.17	0.50–2.74
	*p* for trend						0.91		0.81		0.99	
Overeating	Less than once a week	1686	33.4	19	26.4	15,817	1.00		1.00		1.00	
	2–3 times a week	2864	56.8	43	59.7	27,211	1.26	0.74–2.14	1.22	0.72–2.08	1.24	0.72–2.12
	More than 4 times a week	479	9.5	9	12.5	4525	1.58	0.72–3.48	1.57	0.71–3.48	1.46	0.64–3.31
	*p* for trend						0.20		0.22		0.26	
Added spices	Often	598	11.9	10	13.9	5701	1.00		1.00		1.00	
	Sometimes	2684	53.2	47	65.3	25,530	1.08	0.54–2.13	1.04	0.52–2.06	1.06	0.53–2.12
	Never	1745	34.6	15	20.8	16,305	0.53	0.24–1.19	0.50	0.22–1.11	0.54	0.24–1.20
	*p* for trend						0.04		0.03		0.045	

Abbreviations are as follows: CI, confidence interval; HR, hazard ratio. ^a^ Less than one time a day; ^b^ Adjusted for age, education group (elementary school or less, middle school, high school, college or higher), and breast benign tumor history (yes, no); ^c^ Adjusted for diverse potential confounders: total: age, BMI (<23.0, 23.0 to <25.0, ≥25 kg/m^2^), family history of breast cancer (yes, no), smoking status (current, ex, non-smoker), alcohol consumption (current, ex, non-drinker), physical activity (yes, no), age at menarche (≤13, 14, 15, ≥16 years), parity (0, 1, 2, ≥3), oral contraceptive use (yes, no), benign breast tumor history (yes, no), hormone use and menopausal status (premenopausal, postmenopausal hormone users, postmenopausal hormone non-users, unknown menopausal status), and age at menopause (premenopausal, menopause at <46, 46–48, 49–51, ≥52 years, unknown menopausal status).

**Table 6:** Six dietary habits and their associations with breast cancer risk in premenopausal women.

Dietary Habit	Frequency	Premenopausal Women										
		All (*n* = 2825)		Case (*n* = 48)		Person Years	Age-Adjusted		Multivariate 1 ^b^		Multivariate 2 ^c^	
		*N*	%	*n*	%		HR	95% CI	HR	95% CI	HR	95% CI
Meal frequency	3 times/day	1705	60.4	23	47.9	16,191	1.00		1.00		1.00	
	2 times/day	702	24.9	13	27.1	6527	1.50	0.76–2.96	1.31	0.66–2.61	1.34	0.67–2.68
	Irregular ^a^	400	14.2	12	25.0	3838	2.34	1.16–4.71	2.15	1.06–4.40	2.35	1.13–4.91
	*p* for trend						0.02		0.044		0.03	
Breakfast frequency	Always	1340	47.4	20	41.7	12,623	1.00		1.00		1.00	
	Often	1011	35.8	18	37.5	9604	1.25	0.66–2.36	1.16	0.61–2.20	1.17	0.62–2.24
	Not at all	466	16.5	10	20.8	4414	1.57	0.73–3.38	1.33	0.61–2.92	1.37	0.62–3.05
	*p* for trend						0.24		0.47		0.45	
Meal time regularity	Regular	981	34.7	23	47.9	9162	1.00		1.00		1.00	
	Often	1487	52.6	18	37.5	14,284	0.53	0.28–0.98	0.51	0.27–0.94	0.52	0.28–0.98
	Irregular	348	12.3	7	14.6	3187	0.93	0.40–2.17	0.76	0.32–1.79	0.76	0.31–1.83
	*p* for trend						0.30		0.17		0.19	
Meal speed	Slow	317	11.2	2	4.2	3025	1.00		1.00		1.00	
	Average	1501	53.1	29	60.4	14,138	3.03	0.72–12.71	3.28	0.78–13.77	3.28	0.78–13.84
	Fast	1005	35.6	17	35.4	9536	2.62	0.61–11.35	2.75	0.63–11.93	2.94	0.67–12.86
	*p* for trend						0.49		0.49		0.38	
Overeating	Less than once a week	884	31.3	13	27.1	8257	1.00		1.00		1.00	
	2–3 times a week	1627	57.6	28	58.3	15,466	1.18	0.61–2.27	1.12	0.58–2.17	1.18	0.61–2.30
	More than 4 times a week	304	10.8	7	14.6	2895	1.61	0.64–4.04	1.37	0.54–3.47	1.60	0.62–4.15
	*p* for trend						0.35		0.54		0.37	
Added spices	Often	304	10.8	8	16.7	2854	1.00		1.00		1.00	
	Sometimes	1479	52.4	29	60.4	14,101	0.75	0.34–1.63	0.82	0.37–1.80	0.78	0.35–1.74
	Never	1037	36.7	11	22.9	9719	0.41	0.16–1.01	0.46	0.19–1.16	0.43	0.17–1.08
	*p* for trend						0.03		0.06		0.045	

Abbreviations are as follows: CI, confidence interval; HR, hazard ratio. ^a^ Less than one time a day; ^b^ Adjusted for age, education group (elementary school or less, middle school, high school, college or higher), and breast benign tumor history (yes, no); ^c^ Adjusted for diverse potential confounders: total: age, BMI (<23.0, 23.0 to <25.0, ≥25 kg/m^2^), family history of breast cancer (yes, no), smoking status (current, ex, non-smoker), alcohol consumption (current, ex, non-drinker), physical activity (yes, no), age at menarche (≤13, 14, 15, ≥16 years), parity (0, 1, 2, ≥3), oral contraceptive use (yes, no), and benign breast tumor history (yes, no).

**Table 7:** Six dietary habits and their associations with breast cancer risk in postmenopausal women.

Dietary Habit	Frequency	Postmenopausal Women										
		All (*n* = 2177)		Case (*n* = 23)		Person Years	Age-Adjusted		Multivariate 1 ^b^		Multivariate 2 ^c^	
		*N*	%	*n*	%		HR	95% CI	HR	95% CI	HR	95% CI
Meal frequency	3 times/day	1558	71.6	14	60.9	14,767	1.00		1.00		1.00	
	2 times/day	394	18.1	5	21.7	3668	1.51	0.54–4.24	1.71	0.60–4.81	1.65	0.57–4.76
	Irregular ^a^	208	9.6	4	17.4	1992	2.22	0.73–6.78	1.95	0.64–5.98	1.73	0.53–5.70
	*p* for trend						0.15		0.18		0.28	
Breakfast frequency	Always	1428	65.6	10	43.5	13,511	1.00		1.00		1.00	
	Often	554	25.5	11	47.8	5257	3.05	1.28–7.25	2.86	1.20–6.79	2.98	1.21–7.33
	Not at all	186	8.5	2	8.7	1732	1.75	0.38–8.14	1.58	0.34–7.35	1.83	0.36–9.21
	*p* for trend						0.07		0.10		0.07	
Meal time regularity	Regular	967	44.4	12	52.2	9118	1.00		1.00		1.00	
	Often	1043	47.9	7	30.4	9932	0.56	0.22–1.44	0.62	0.24–1.60	0.60	0.23–1.58
	Irregular	158	7.3	4	17.4	1442	2.20	0.70–6.94	3.10	0.96–9.99	2.28	0.64–8.10
	*p* for trend						0.76		0.49		0.70	
Meal speed	Slow	238	10.9	5	21.7	2195	1.00		1.00		1.00	
	Average	1116	51.3	10	43.5	10,533	0.43	0.15–1.24	0.52	0.18–1.54	0.46	0.15–1.42
	Fast	820	37.7	8	34.8	7827	0.46	0.15–1.41	0.56	0.18–1.72	0.45	0.13–1.54
	*p* for trend						0.30		0.42		0.30	
Overeating	Less than once a week	793	36.4	6	26.1	7477	1.00		1.00		1.00	
	2–3 times a week	1203	55.3	14	60.9	11,426	1.38	0.55–3.46	1.34	0.54–3.37	1.23	0.47–3.24
	More than 4 times a week	174	8.0	2	8.7	1618	1.40	0.29–6.83	1.47	0.30–7.14	1.11	0.21–5.92
	*p* for trend						0.38		0.36		0.52	
Added spices	Often	289	13.3	2	8.7	2797	1.00		1.00		1.00	
	Sometimes	1182	54.3	17	73.9	11,214	2.20	0.51–9.52	1.93	0.44–8.49	2.47	0.54–11.28
	Never	692	31.8	4	17.4	6437	0.91	0.17–4.95	0.79	0.14–4.40	1.14	0.20–6.60
	*p* for trend						0.50		0.41		0.73	

Abbreviations are as follows: CI, confidence interval; HR, hazard ratio. ^a^ Less than one time a day; ^b^ Adjusted for age, education group (elementary school or less, middle school, high school, college or higher), and breast benign tumor history (yes, no); ^c^ Adjusted for diverse potential confounders: total: age, BMI (<23.0, 23.0 to <25.0, ≥25 kg/m^2^), family history of breast cancer (yes, no), smoking status (current, ex, non-smoker), alcohol consumption (current, ex, non-drinker), physical activity (yes, no), age at menarche (≤13, 14, 15, ≥16 years), parity (0, 1, 2, ≥3), oral contraceptive use (yes, no), benign breast tumor history (yes, no), hormone use (yes, no), and age at menopause (<46, 46–48, 49–51, ≥52 years).

## Appendix A

Food list for the 16 food groups in the short-form FFQ:	
Food group	Food list
Cereals	Rice, bread, noodles, potatoes, sweet potatoes
Salted vegetables and seafood	Kimchi, salted fermented seafood, food boiled in soya with spices
Light-colored vegetables	Bean sprouts, cucumbers, radishes, onions, bellflower
Green-yellow vegetables	Carrots, spinach, sesame leaf, lettuce, zucchini, dropwort
Seaweed	Laver, brown seaweed, tangle
Fruit	Apples, tangerines, grapes, watermelon, strawberries, peaches, pears, fruit juice
Grilled meat	Grilled ribs, barbecue
Healthy protein foods	Lean meat, fish, legumes, soy foods (e.g., soybean curd), egg whites
Dairy foods	Yogurt, cheese, milk
Bony fish	Anchovies, dried whitebait
Fried foods	Deep-fried food, stir-fried food
High-cholesterol foods	Egg yolks, seafood (eel, shrimp, squid), organ meats (from fish or other animals)
Animal fat-rich foods	High-fat red meat (beef, pork), processed food (ham, sausage), butter
Sweet foods	Bread, cookies, chocolate, honey, candy, ice cream
Fast foods	Pizza, chicken, processed food
Caffeinated drinks	Coffee, black tea, cocoa, coke
